# New rare genetic variants in multiple sclerosis

**DOI:** 10.1007/s00415-018-9128-9

**Published:** 2018-11-20

**Authors:** Katharine E. Harding, Neil P. Robertson

**Affiliations:** Department of Neurology, Institute of Psychological Medicine and Clinical Neurosciences, University Hospital of Wales, Cardiff University, Heath Park, Cardiff, CF14 4XN UK

## Introduction

In recent years, our understanding of genetic factors associated with multiple sclerosis (MS) has considerably increased as a result of new analytical approaches to genome-wide association studies, together with the large numbers of samples available through international collaborations. It is now clear that MS risk is multigenic, and that common variants (now numbering more than 150) contribute only a relatively small proportion to the overall heritability of MS. As a result, attention has once again turned to the possibility of identifying rare variants through a number of novel as well as more traditional methodologies. However, detecting effects of rare variants once again poses challenges of statistical power, and several different approaches can be applied to manage this issue, some of which are illustrated below. In this month’s Journal Club we review five recent publications that have employed large international cohorts, multiplex families, or candidate gene approaches to investigate rare genetic variants and their association with MS risk.

## Low-frequency and rare-coding variation contributes to multiple sclerosis risk

In this study, the International Multiple Sclerosis Genetics Consortium (IMSGC) used exome arrays from a total of 36,219 MS cases and 38,629 controls from 12 countries to investigate rare variants in MS. Mixed linear models incorporating genotypic principal components were used to analyse association with disease. Seven rare variants were identified in six genes, although two variants were in linkage disequilibrium with common variants already known to be associated with MS (*TYK2* and *GALC*). The other variants were in genes not previously known to be associated with MS, but which are involved in the innate (*NLRP8*) and adaptive immune systems (*HDAC7, PRF1*, and *PRKRA*), and therefore, are plausible candidates for pathogenesis of MS. Interestingly, the minor alleles were protective in most cases, which is the reverse to that which is usually found with common variants.

*Comment*. This is an interesting and important study. The IMSGC has been very successful over the last 15 years in using genome-wide association studies to identify common variants associated with MS, and have now used newer genetic techniques in their established large cohort of patients to detect rare variants. This study adds to our knowledge about the genetic factors in MS pathogenesis. However, the rare variants identified contribute approximately 5% of MS heritability (in addition to the 19% heritability contributed by common variants). This suggests that other variants also contribute to the genetic heritability of MS and that larger studies or alternative approaches may be required to discover them. In addition, more work is needed to understand the mechanisms by which the genes identified in this study may be relevant for MS pathogenesis.

International Multiple Sclerosis Genetics Consortium (2018) Cell. 10.1016/j.cell.2018.09.049 (epub ahead of print).

## Genome sequencing uncovers phenocopies in primary progressive multiple sclerosis

This study investigated genetic variation in primary progressive MS (PPMS), with the hypothesis that genes known to cause diseases that are phenotypically similar to PPMS may also be relevant in PPMS pathogenesis. Specifically, this study focused on genes known to be associated with hereditary spastic paraplegia (HSP). Whole genome sequencing data was used in samples from 38 PPMS patients and 81 controls, and pathogenic variants known to occur in monogenic neurological disorders that were observed only in the PPMS patients were then sequenced in two replication cohorts comprising a total of 746 PPMS, 3049 relapsing-onset MS, and 1000 healthy controls. Additionally, the genetic burden of variants in genes associated with HSP was analysed in PPMS, relapsing-onset MS and healthy controls. Three variants were identified in PPMS patients: *KIF5A* (associated with spastic paraplegia 10), *MLC1* (associated with megalencephalic leukodystrophy with subcortical cysts), and *REEP1* (associated with spastic paraplegia 31), and the burden of genetic variants associated with HSP was higher in PPMS patients than controls.

*Comment*. Given the logistic challenges involved in detecting rare variants in MS, as illustrated by the previous paper, the approach taken in this study of targeting genes known to cause syndromes phenotypically similar to MS seems logical. Focussing on PPMS is an important aspect of this study, as it remains a less well-understood MS phenotype, and an improved understanding of pathogenesis may also lead to improved management and therapeutic strategies. Although the discovery cohort in this study was relatively small, the two-stage validation approach on larger cohorts of patients increases plausibility and robustness of the findings.

Jia X, Madireddy L, Caillier S et al (2018) Ann Neurol 84(1):51–63.

## Common genetic etiology between “multiple sclerosis-like” single gene disorders and familial multiple sclerosis

This study hypothesised that genes causing diseases phenotypically similar to MS may also contain rare variants that are relevant for MS pathology. To investigate this, samples from a cohort of patients from British Columbia, Canada were used to examine rare variants in candidate genes as follows: 28 candidate genes were identified. Exome sequencing was available in 270 MS cases, and this was used to identify 57 rare variants present in the candidate genes. These variants were then genotyped in 830 MS cases and 2131 controls to identify which variants occurred in MS cases but not controls. Finally, where family samples were also available, the remaining variants were analysed to look for segregation with the disease within families. This process identified a total of 9 variants in 6 genes, three of which were all seen within one multi-incident family (*CLCN2, GALC*, and *POLG*). The other three genes identified were *CYP27A1, LYST* and *PDHA1*.

*Comment*. The variants identified in this study are involved in cholesterol metabolism (*CYP27A1*) or mitochondrial function (*POLG* and *PDHA1*), suggesting additional mechanisms for pathogenesis of MS. This study also demonstrates that even within a single family there may be more than one genetic variant that could be relevant, and underlines the multigenic nature of MS and hints at further reasons for variability of outcome in MS.

Traboulsee AL, Sadovnick AD, Encarnacion M et al (2017) Hum Genet 136:705–714

## Linkage analysis and whole exome sequencing identify a novel candidate gene in a Dutch multiple sclerosis family

This study investigated a large Dutch family with 11 affected individuals as well as unaffected individuals including obligate carriers. The initial genome linkage analysis identified a potential region of interest on chromosome 7. Exome sequencing was then carried out on four affected family members which identified eight candidate rare variants. These variants were then genotyped in the whole family, and a single variant was found to segregate with disease located in the *FKBP6* gene. However, no difference was found in the frequency of the variant when typed in 591 sporadic MS cases compared to healthy controls. *FKBP6* is a receptor involved in immunological function and prediction of structural effects of the variant suggests that it is likely to be damaging to the protein’s function.

*Comment*. In a similar fashion to other papers reviewed in this article, this study has identified a rare variant in a gene that has also been implicated in other genetic disorders; in this case Williams–Beuren syndrome which is associated with cardiovascular defects, learning difficulties and hypercalcaemia. This finding has not previously been reported in MS. Although this variant may be important for the pathogenesis of MS in this family, the fact that the variant was no different in cases compared to controls limits its overall relevance to MS.

Mescheriakova JY, Verkerk AJMH, Amin N et al (2018) Mult Scler 1352458518777202. 10.1177/1352458518777202 (epub ahead of print).

## Purinergic receptors P2RX4 and P2RX7 in familial multiple sclerosis

This study used exome sequencing data from the British Columbia patient cohort and a family with multiple affected members to investigate related candidate genes: The purinergic receptors *P2RX7* and *P2RX4* are transmembrane cation channels known to be important in the pro-inflammatory cascade, and have previously been shown to modulate MS susceptibility. A rare three-variant haplotype in the *P2RX7* and *P2RX4* genes was identified which segregated with MS within the family. The authors then went on to a functional analysis of the variants, and were able to demonstrate impaired channel function, which led to a substantial reduction in phagocytic ability of the cell.

*Comment*. One of the key features of this work is its investigation of functional effects of newly described rare variants. This is clearly a necessary step to understand the potential biological role of these genetic variations and how they might lead to immune system dysfunction and/or precipitate the pathology known to be associated with MS. It also illustrates the value of family studies in identifying genetic variants associated with MS, although again it may be the case that these variants are less important in sporadic MS. Finally, the British Columbia cohort has a very high standard of clinical phenotyping and the two studies here described from this cohort illustrate the vital importance of detailed clinical phenotype for genetic studies.

Sadovnick AD, Gu BJ, Traboulsee AL et al (2017) Hum Mutat 38(6):736–744.

